# Exploring the Relationship Between Excessive Social Media Use and Eating Disorders Among Young Adults: Evidence From a Bangladesh‐Based Cross‐Sectional Study

**DOI:** 10.1002/brb3.70540

**Published:** 2025-05-11

**Authors:** Trisha Mallick, Md. Hasan Al Banna, Tasnim Rahman Disu, Shammy Akter, Tareq Mahmud, Tasnima Akhter Tasin, Nargees Akter, Md. Saiful Alam, Md. Nazmul Hassan

**Affiliations:** ^1^ Department of Environmental Sanitation, Faculty of Nutrition and Food Science Patuakhali Science and Technology University Patuakhali Bangladesh; ^2^ Department of Food Microbiology, Faculty of Nutrition and Food Science Patuakhali Science and Technology University Patuakhali Bangladesh; ^3^ Institute of Public Health Nutrition Mohakhali, Dhaka Bangladesh; ^4^ Department of Applied Nutrition and Food Technology, Faculty of Biological Sciences Islamic University Kushtia Bangladesh; ^5^ Department of Public Health and Informatics Jahangirnagar University Savar Bangladesh; ^6^ Department of Geography and Environmental Studies University of Chittagong Chittagong Bangladesh

**Keywords:** Bangladesh, eating disorders, Facebook addiction, young adults

## Abstract

**Objectives:**

Problematic or addictive use of social media has been associated with psychological and health issues. The main objective of this study was to explore the relationship between Facebook addiction and eating disorders (EDs) among young adults in Bangladesh.

**Methods:**

We conducted an online‐based cross‐sectional study among 550 young adults aged 18 to 27 in Bangladesh. A structured questionnaire was used to obtain the survey data. The survey tool consisted of three sections: (i) socio‐demographic, behavioral, and social media use‐related characteristics, (ii) assessment of Facebook addiction using Bergen Facebook Addiction Scale, and (iii) assessment of EDs risk using Eating Attitudes Test‐26 (EAT‐26, outcome variable). Scoring at or above 20 on the EAT‐26 scale (total score ranges from 0 to 78) indicated an ED risk. Unadjusted and adjusted binary logistic regression analyses were performed to investigate the relationship outcome and explanatory variables.

**Results:**

Approximately 38% of the study participants showed addiction to Facebook, whereas 23.6% were at risk of developing an ED. Multiple adjusted logistic regression models demonstrated that Facebook addiction was significantly associated with an increased risk of EDs (OR  =  1.784; 95% confidence interval [CI]: 1.154–2.760). Moreover, smoking habits, self‐rated body mass index (BMI), and physical activity level showed a significant association with the risk of EDs.

**Conclusions:**

These findings may help public health professionals and policymakers to take the initiative and develop strategies to overcome these addictive behaviors and promote healthy eating habits across the country.

## Graphical abstract

This cross‐sectional study (*n* = 550) revealed a significant association between Facebook addiction and increased eating disorder risk. These findings highlight the need for public health interventions to address addictive behaviors and promote healthy eating habits in Bangladesh.

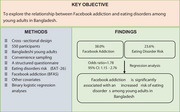

## 1 Introduction

Eating disorders (EDs) are characterized by inappropriate eating behavior and have negative impacts on both physical and mental health (Alsheweir et al. 2023). According to the World Health Organisation (WHO) and the American Psychiatric Association (APA), the most prevalent EDs worldwide are bulimia nervosa (BN), anorexia nervosa (AN), and binge ED (BED) (Alsheweir et al. 2023). People who have been diagnosed with AN (severe weight loss and limited food intake) or BN (massive food intake followed by vomiting) try to reach or maintain a low body weight because they have misconceptions about their physical appearance (Eskander et al. 2020; Keel 2017).

EDs are associated with the highest rate of mortality among all psychological disorders (Streatfeild et al. 2021; van Hoeken and Hoek 2020). Young adults and adolescents are the most at risk of disordered eating behavior (Alsheweir et al. 2023), and it is significantly impacting their lives (Rikani et al. 2013). The early stages of adulthood related to various social responsibilities may have distinct impacts on eating habits and lifestyle for both men and women. Compared to young men, young women express more risky attitudes and disordered eating behavior (Mayer et al. 2004).

EDs are on the increase throughout Asia and the Pacific regions due to various key cultural transformations, including economic development, urbanization, and fundamental alterations in gender roles, family life, and dietary habits (Grilo 2024; Kim et al. 2021; Li et al. 2021; Thomas et al. 2016). According to estimates ranging from 20.4% to 38%, the risk of ED has been rising among Bangladeshi young adults (university students) (Banna et al. 2025). A recent Bangladeshi review study summarized that university students’ ED risk is associated with the following factors: age, sex, academic attainment, marital status, family income, smoking, nutritional status, anxiety, depression, internet addiction, high religious practice, previous cosmetic surgery, and binge drinking (Banna et al. 2025). Furthermore, several social, cultural, and psychological aspects affect eating attitudes and habits (He et al. 2022). A study found a relationship between Facebook addiction and eating habits among young adults (Filippone et al. 2022). Increased exposure to Western beauty standards and lifestyle content on social media has been associated with the adoption of disordered eating habits and unhealthy lifestyle patterns among young adults as they are the most vulnerable group to be addicted to social media (Fardouly et al. 2015; Holland and Tiggemann 2016; Sidani et al. 2016).

In Bangladesh, approximately 52.90 million people used social media in 2024, of which 31.4 million were Facebook users, with young adults (aged 18–24 years) representing the largest user group (Cat 2024; DataReportal 2024). Facebook is generally considered one of the world's most popular social networking sites, with a substantial influence on interpersonal communications (Pang et al. 2021). However, problematic usage emerges when involvement becomes excessive and uncontrollable, leading to impairment in daily functioning, interpersonal relationships, and psychological well‐being (Marino et al. 2018; Schou Andreassen and Pallesen 2014). Previous studies (Al‐Mamun et al. 2022; Karim et al. 2023; Sayeed et al. 2023) indicate that problematic Facebook use or Facebook addiction among Bangladeshi young adults shows a concerning prevalence rate ranging from 29.4% to 37.1%. A longitudinal study observed “maladaptive” Facebook addiction and changes in eating habits for more than 4 weeks among college women and reported that Facebook addiction had a negative impact on their eating habits (Smith et al. 2013). Additionally, watching videos generated by social media influencers on various social networking sites, such as Facebook, influences individual eating patterns (Cha EunMe et al. 2018).

However, in Bangladesh, there have not been any studies conducted on the relationship between Facebook addiction and EDs among young adults. Exploring this particular relationship is justified by the following possible reasons: (i) Due to Facebook's wide availability, ability to serve multiple purposes, and active user bases, it has become more popular than other platforms like Instagram, TikTok, and X in Bangladesh. Consequently, Facebook provides a more representative platform to investigate young people's usage of social media and their behavioral consequences. (ii) As the prevalence of both EDs (Banna et al. 2025) and Facebook addiction (Sayeed et al. 2023) among young adults is increasing in the country, revealing the correlational link between Facebook addiction and EDs would provide baseline evidence to inform policy and intervention initiatives.

The main objective of this study was to assess the relationship between Facebook addiction and the risk of ED among a sample of Bangladeshi young adults.

## 2 Materials and Methods

### 2.1 Study Design and Participants

A cross‐sectional online survey was performed between November 2023 and January 2024 among young adults in Bangladesh. People who use Facebook or have a Facebook account from all over Bangladesh made up the sample. The inclusion criteria for the study participants were as follows: (i) being born in Bangladesh and living there during the survey period; (ii) being at least 18 years old; and (iii) having a Facebook account. Individuals who do not use Facebook or do not have any Facebook account, even if they use other social media platforms such as Instagram, X, TikTok, or others, were excluded.

### 2.2 Sample Size Calculation

We utilized a single sample proportion test to determine the minimum number of samples required. Our estimated minimum required sample (*n*) was 360 participants by considering *d* = margin of error (5%), *Z* = 95% confidence interval (CI) (1.96), and *p* = 37.6% overall prevalence of ED among university students of Bangladesh (Pengpid et al. 2015). The equation was as follows:

$$\begin{array}{ccc} \mathrm{The}\;\mathrm{minimum}\;\mathrm{sample},\;n & = & \frac{z^{2}\times p\times (1-p)}{d^{2}}\\ & & =\frac{{(1.96)}^{2}\times 0.376\times (1-0.376)}{{(0.05)}^{2}}\\ & & =360.53\approx 360. \end{array}$$

Thus, a total of 651 people from throughout Bangladesh took part in the survey. We then eliminated data that was missing or ineligible, and the final sample size for this research consisted of 550 respondents.

### 2.3 Sampling and Survey Procedures

We used convenience sampling to enroll the participants. An online survey through a Google Form was used to gather the information. It was first written in English, then translated into Bengali by the study team, and the lead researcher checked it again. Once it was completed, four research assistants shared the Google Form link with their networks on social media sites, including Facebook, WhatsApp, Instagram, and others.

Participants were directed to the survey front pages, which had an informed consent page, a short description of the research, and instructions on how to complete the form after clicking on the survey link. It was also clarified that the data gathered would only be used for research purposes, and participation in the study was completely voluntary. To ensure data privacy and maintain confidentiality of the participants, the data were kept in a password‐protected folder.

### 2.4 Measures

The survey consisted of three parts: (i) socio‐demographic, behavioral, and social media use‐related characteristics; (ii) Facebook addiction using the Bergen Facebook Addiction Scale (BFAS); and (iii) assessment of ED using Eating Attitudes Test‐26 (EAT‐26).

#### 2.4.1 Socio‐Demographic, Behavioral, and Social Media Use‐Related Information

Participants’ socioeconomic characteristics, including sex, age, marital status, type of residence, size of family, level of education, occupation, and monthly income, were obtained in the first part of the questionnaire. Data on behavior and social media use included the following: smoking habits (yes vs. no), self‐rated body mass index (BMI) status (underweight, normal body weight, or overweight/obesity), level of physical activity (physically inactive, moderate activity level, or regular activity level), and Facebook using time (≤to 3 h, 4–7 h, or >7 h) per day. Participants’ level of physical activity was measured using methods consistent with previous Bangladeshi studies (Abid et al. 2023; Sahrin et al. 2024).

#### 2.4.2 Bergen Facebook Addiction Scale (BFAS)

The BFAS (Ahmed and Hossain 2018; Andreassen et al. 2012) was derived from the Bergen Social Media Addiction Scale, which was modified by replacing the term “social media” with “Facebook.” The BFAS has six questions with a 5‐point Likert scale (e.g., “How often during the last year have you felt an urge to use Facebook more and more?” and “How often during the last year have you used Facebook to forget about personal problems?”) with scores varying from very rarely (1) to very often (5). These questions determined symptoms that occurred throughout the last 12 months. The total score varies from 6 to 30. A greater likelihood of Facebook addiction was indicated by a higher score. According to the monothetic scoring scheme, we used a cutoff score of ≥18 in this study, though the original study did not use any cutoff value (Al Mamun and Griffiths 2019; Schou Andreassen and Pallesen 2014). This cutoff score was previously used in the studies conducted in Bangladesh (Sayeed et al. 2020, 2023). The BFAS has demonstrated strong validity and reliability across multiple studies (Al Mamun and Griffiths 2019; Atroszko et al. 2018; Brailovskaia et al. 2018). Ahmed and Hossain (2018) established the scale's (BFAS) reliability and validity in measuring Facebook addiction in Bangladesh. The Cronbach's alpha for the BFAS was 0.74 in the current study, indicating an adequate degree of internal reliability.

#### 2.4.3 ED Risk (EAT‐26)

The risk of ED (outcome variable) was measured using a validated EAT‐26 scale that consisted of three subscales with 26 items of statements. A six‐point Likert scale from “always” to “never” was used to assess the 3 subscales, such as Factor I, which is “dieting” with 13 statements; Factor II, which is “bulimia and food preoccupation” with 6 statements; and Factor III, which is “oral control” with 7 statements. For every 26 statements, participants received a response, such as “I am terrified about being overweight,” “I avoid eating when I am hungry,” and so on. The responses for “always,” “usually,” and “often” received scores of 3, 2, and 1, respectively, whereas the responses for “sometimes,” “rarely,” and “never” received scores of 0 (inverse scoring for item 26). To determine the risk of ED, a cutoff score ≥20 was considered “at risk,” and a score of <20 indicated “no risk” (the total score ranges from 0 to 78) (Pengpid et al. 2015; Banna et al. 2021).

### 2.5 Statistical Analysis

For analyzing the data, we used IBM SPSS for Windows, version 23.0. Descriptive analysis, including frequencies, percentages, means, and standard deviations (SD), was applied to summarize the data. The Chi‐square test was done to compare the EDs (at‐risk vs. no‐risk) by participants’ socio‐demographic, behavioral, and social media use‐related characteristics. Moreover, the Spearman correlation analysis was performed to observe the association between EAT‐26 and BFAS scores. In this study, the outcome variable was dichotomous; hence, a binary logistic regression model was fitted to identify the factors associated with ED risk. In the unadjusted binary logistic regression model, crude odds ratio (COR) with a corresponding 95% CI was calculated for all the independent variables. Before running the adjusted binary regression model, all independent variables were verified for multicollinearity using the variance inflation factor, and no multicollinearity was detected. A backward binary logistic regression approach was used, where the model commences with all independent variables (saturated model) and progressively excludes the less influential variables from the saturated model. The adjusted model was fitted on the basis of the Hosmer and Lemeshow goodness‐of‐fit tests, and the level of significance was specified at *p* values less than 0.05.

### 2.6 Ethics Consideration

The Institutional Ethical Committee of Patuakhali Science and Technology University (Bangladesh) has given the ethical clearance [approval number: PSTU/IEC/2023/71]. Informed consent (e‐consent) was acquired from the participants.

## 3 Results

### 3.1 Sample Characteristics and Descriptive Data

The final sample of this study constituted 550 young adults with a mean age of 22.78 years (SD ±2.059 and ages ranged from 18 to 27 years). More than half of the participants were male (54.5%). A higher proportion of the participants (57%) lived in city areas. Approximately one‐third of the respondents (33.1%) reported abnormal body weight (either underweight or overweight/obese). Above half of them had moderate physical activity levels (55.1%), and 17.5% had smoking habits. On the basis of the BFAS (mean score 15.51, SD ±4.682), more than one‐third of the participants had Facebook addiction (38.0%). Detailed information on participants’ socio‐demographic, behavioral, and social media use‐related characteristics is presented in Table 1.

**TABLE 1:** Socio‐demographic, behavioral, and social media use‐related characteristics of study participants (*n* = 550).

Variables	Categories	Frequency	Percentage (%)
**Gender**	Male	300	54.5
	Female	250	45.5
**Age**	18–20 years	73	13.3
	21–24 years	381	69.3
	25–27 years	96	17.5
**Marital status**	Single	485	88.2
	Married	65	11.8
**Residency type**	City area	317	57.6
	Sub‐urban area	148	26.9
	Rural area	85	15.5
**Family size**	≤5 members	395	71.8
	>5 members	155	28.2
**Education level**	Intermediate or below	61	11.1
	Bachelor	400	72.7
	Higher education (above bachelor)	89	16.2
**Monthly income**	≤20,000 BDT (Bangladeshi taka)	183	33.3
	21,000–40,000 BDT	226	41.1
	>40,000 BDT	141	25.6
**Smoking habits**	Yes	96	17.5
	No	454	82.5
**Self‐rated BMI**	Underweight	76	13.8
	Normal weight	368	66.9
	Overweight/obesity	106	19.3
**Physical activity level**	Physically inactive	25	4.5
	Moderate activity level	303	55.1
	Regular activity level	222	40.4
**Facebook using time per day**	≤3 h	169	28.9
	4–7 h	270	49.1
	>7 h	121	22.0
**Facebook addiction**	No	341	62.0
	Yes	209	38.0

### 3.2 Prevalence and Predictors of EDs Risk

Approximately 23.6% of the study participants were at risk of ED. In Table 2, the Chi‐square test showed that five variables, such as participant's marital status (*p* = 0.018), residency types (*p* = 0.043), self‐rated BMI (*p* = 0.000), physical activity level (*p* = 0.002), and Facebook addiction (*p* = 0.009), were strongly associated with ED.

**TABLE 2:** Presentation of eating disorders (at‐risk vs. no risk) by participants’ socio‐demographic, behavioral, and social media use‐related characteristics.

	Categorization of EAT‐26		Chi‐square test	
Variables	No risk of eating disorders	At‐risk of eating disorders	Value (df)	*p* value
**Gender**				
Male	232 (77.3%)	68 (22.7%)	0.344 (1)	0.558
Female	188 (75.2%)	62 (24.8%)		
**Age**				
18–20 years	57 (78.1%)	16 (21.9%)	0.138 (2)	0.933
21–24 years	290 (76.1%)	91 (23.9%)		
25–27 years	73 (73.3%)	23 (24.0%)		
**Marital status**				
Single	378 (77.9%)	107 (22.1%)	5.637 (1)	**0.018**
Married	42 (64.6%)	23 (35.4%)		
**Residency type**				
City area	230 (72.6%)	87 (27.4%)	6.305 (2)	**0.043**
Sub‐urban area	119 (80.4%)	29 (19.6%)		
Rural area	71 (83.5%0	14 (16.5%)		
**Family size**				
≤5 members	304 (77.0%)	91 (23.0%)	0.278(1)	0.598
>5 members	116 (74.8%)	39 (25.2%)		
**Education level**				
Intermediate or below	40 (65.6%)	21 (32.4%)	5.186 (2)	0.075
Bachelor	314 (78.5%)	86 (21.5%)		
Higher education (above bachelor)	66 (74.2%)	23 (25.8%)		
**Monthly income (Bangladesi taka, BDT)**				
≤20,000 BDT	135 (74.6%)	48 (25.4%)	1.165 (2)	0.559
21,000–40,000 BDT	177 (79.6%)	49 (20.4%)		
>40,000 BDT	108 (74.5%)	33 (25.5%)		
**Smoking habits**				
Yes	67 (69.8%)	29 (30.2%)	2.783 (1)	0.095
No	353 (77.8%)	101 (22.2%)		
**Self‐rated BMI**				
Underweight	69 (90.8%)	7 (9.2%)	38.442 (2)	**0.000**
Normal weight	293 (79.3%)	75 (20.4%)		
Overweight/obesity	58 (56.9%)	48 (45.3%)		
**Physical activity level**				
Physically inactive	12 (48.0%)	13 (52.0%)	12.195 (2)	**0.002**
Moderate activity	232 (76.6%)	71 (23.4%)		
Regular activity	176 (79.3%)	46 (20.7%)		
**Facebook using time per day**				
≤3 h	127 (79.9%)	32 (20.1%)	5.431 (2)	0.066
4–7 h	210 (77.8%)	60 (22.2%)		
>7 h	83 (68.6%)	38 (31.4%)		
**BFAS**				
No	273 (80.1%)	68 (19.9%)	6.788 (1)	**0.009**
Yes	147 (70.3%)	62 (29.7%)		

*Note*: Bolded value indicates statistically significant (*p* < 0.05).

**FIGURE 1:**
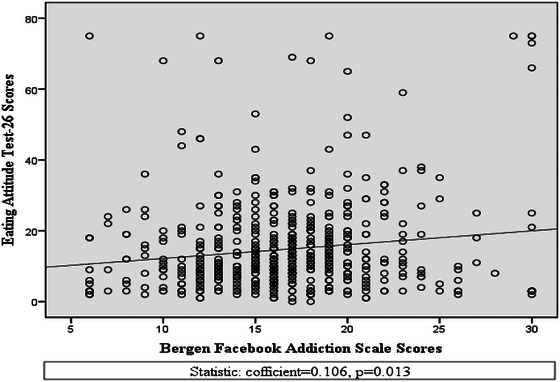
Simple scatter plot depicting the positive significant correlation between EAT‐26 and BFAS scores.

Moreover, Spearman correlation analysis found a weak but statistically significant positive correlation between EAT‐26 and BFAS scores (Spearman coefficient, *r_s_* = 0.106, *p* = 0.013) (Figure 1).

In Table 3, the results of unadjusted binary regression analysis demonstrated that the following factors were associated with the risk of ED: (i) being single (COR  = 0.517; 95% CI: 0.298–0.898), (ii) being residence at city area (COR =  1.918; 95% CI: 1.028–3.580), (iii) being underweight (COR  = 0.39; 95% CI: 0.175–0.898) and overweight/obese (COR =  3.233; 95% CI: 2.043–5.116), (iv) being physically inactive (COR =  4.145; 95% CI: 1.773–9.689), (v) >7 h of Facebook use (COR =  1.817; 95% CI: 1.053–3.135), and (vi) Facebook addiction (COR =  1.693; 95% CI: 1.137–2.522).

**TABLE 3:** Unadjusted binary logistic regression estimate demonstrating the predictors of eating disorder risk among young adults in Bangladesh.

	Unadjusted binary regression model			
	Odds ratio	95% confidence interval		*p* value
Variables		Lower value	Upper value	
**Gender**				
Male Female	0.889	0.599	1.318	0.558
	Reference			
**Age**				
18–20 years	0.891	0.431	1.841	0.755
21–24 years	0.996	0.589	1.683	0.988
25–27 years	Reference			
**Marital status**				
Single	0.517	0.298	0.898	**0.019**
Married	Reference			
**Residency type**				
City area	1.918	1.028	3.580	**0.041**
Sub‐urban area	1.236	0.612	2.495	0.554
Rural area	Reference			
**Family size**				
≤5 members	0.890	0.578	1.371	0.598
>5 members	Reference			
**Education level**				
Intermediate or below	1.507	0.741	3.064	0.258
Bachelor	0.786	0.462	1.337	0.374
Higher education	Reference			
**Monthly income**				
≤20,000 BDT	Reference			
21,000–40,000 BDT	0.779	0.493	1.229	0.283
>40,000 BDT	0.859	0.516	1.432	0.561
**Smoking habits**				
Yes	1.513	0.928	2.466	0.097
No	Reference			
**Self‐rated BMI**				
Underweight	0.396	0.175	0.898	**0.027**
Normal weight	Reference			
Overweight/obesity	3.233	2.043	5.116	**0.000**
**Physical activity level**				
Physically inactive	4.145	1.773	9.689	**0.001**
Moderate activity level	1.171	0.770	1.781	0.461
Regular activity level	Reference			
**Facebook using time per day**				
≤3 h	Reference			
4–7 h	1.134	0.700	1.837	0.610
>7 h	1.817	1.053	3.135	**0.032**
**BFAS**				
No	Reference			
Yes	1.693	1.137	2.522	**0.010**

*Note*: Bolded value indicates statistically significant (*p* < 0.05).

In Table 4, we presented the factors correlated with the risk of ED among study participants after adjusting the variables. The adjusted regression model was fitted by the Hosmer and Lemeshow test (Chi‐square = 3.868, df = 7, *p* = 0.795). The findings from adjusted regression analysis showed that the risk of developing ED was higher among young adults who had smoking habits compared to non‐smoker counterparts (adjusted odds ratio, AOR = 1.726; 95% CI: 1.002–2.907). Those young adults who were overweight or obese had a two times higher possibility of having a risk of ED as compared to normal weight (AOR = 2.777; 95% CI: 1.656–4.656). The risk of having an ED was three times higher among young adults who were physically inactive compared to their counterparts (AOR =  3.891; 95% CI: 1.535–9.861). Young adults who were addicted to Facebook (according to BFAS) had a higher risk of developing EDs compared to those who were not addicted to Facebook (AOR =  1.784; 95% CI: 1.154–2.760).

**TABLE 4:** Multiple binary logistic regression model (backward model) showing the statistically significant factors of eating disorder risk among young adults in Bangladesh.

Variables	Adjusted model			
	Odds ratio	95% confidence interval		*p* value
		Lower value	Upper value	
**Marital status**				
Single	0.579	0.313	1.071	0.081
Married	Reference			
**Smoking habits**				
Yes	1.726	1.002	2.907	**0.040**
No	Reference			
**Self‐rated BMI**				
Underweight	0.310	0.132	0.730	**0.007**
Normal weight	Reference			
Overweight/obesity	2.777	1.656	4.656	**0.000**
**Physical activity level**				
Physically inactive	3.891	1.535	9.861	**0.004**
Moderate activity level	0.851	0.534	1.356	0.498
Regular activity level	Reference			
**Facebook addiction**				
No	Reference			
Yes	1.784	1.154	2.760	**0.009**

*Note*: Variables entered on the saturated model: gender, age, marital status, residency type, family size, education level, monthly income, smoking habits, self‐rated BMI, physical activity level, social media using time per day, and BFAS. Bolded value indicates statistically significant (*p* < 0.05).

## 4 Discussion

### 4.1 Key Findings

This study examined the relationship between Facebook addiction and ED risk among young adults in Bangladesh. The finding shows that Facebook addiction was significantly associated with ED risk. Moreover, study participant's smoking habits (being a smoker), self‐rated BMI (being overweight/obese), and physical activity level (being physically inactive) were found to have a greater risk of ED.

### 4.2 Prevalence of ED Risk Among Young Adults

Our research showed that 23.6% of young adults were at risk for ED, which allings from findings in Bangladesh and Pakistan (Memon et al. 2012; Banna et al. 2021). However, prior studies among university students reported a lower prevalence of ED risk in India (4%) (Balhara et al. 2012) and China (2.5%) (Liao et al. 2010) compared to the current study. This elevated risk for ED may be linked to greater Western cultural influence, shifting societal norms, and globalization—including urbanization and digital media exposure—which promote thinness ideals and encourage increased food or beverage consumption through targeted advertising (Pengpid et al. 2015; Pike and Dunne 2015). The most vulnerable group for ED in terms of attitudes and behaviors are young adults, especially university students who are also referred to as emerging adulthood (Smink et al. 2012). During this time, most of them live outside of their homes and start university, and their lack of understanding about maintaining a healthy lifestyle, particularly in terms of eating behaviors, contributes to the risk of many mental and disordered eating behaviors (Auerbach et al. 2016; Buyuktuncer et al. 2018; Ibrahim et al. 2013). The symptoms of ED, particularly in young adults, have gathered attention from throughout the world (Alpaslan et al. 2015) as behavioral patterns and lifestyle are often developed during this period (Çelik et al. 2015).

### 4.3 Relationship Between Facebook Addiction and Risk of ED

The prevalence of Facebook addiction in the present study was 38% that was higher than past study conducted in Bangladesh (Sayeed et al. 2020). We found a statistically significant positive correlation between BFAS and EAT‐26 scores (*r_s_* = 0.106, *p* = 0.013); however, the effect size shows only a weak association between these variables. On the basis of Cohen's (1988) conventional standards for interpreting correlation coefficients (i.e., 0.10 = small, 0.30 = medium, and 0.50 = large), the implication of this finding is that although there was indeed a quantifiable relationship between Facebook addiction and ED risk, the shared variance between these variables is relatively small. The clinical significance of this relationship can thus be limited, even if the consistent direction of association in our analyses warrants further investigation.

The adjusted binary logistic regression model showed young adults who had Facebook addiction were more likely to be a risk of ED than non‐addicted participants. Previous studies have also shown that using Facebook may contribute to disordered eating behaviors (Stronge et al. 2015; Walker et al. 2015). Moreover, social media is mostly used by young adults to communicate, have fun, and improve their careers, which makes it essential for university students (Ghamari et al. 2011). An individual who uses social media sites such as Facebook is more likely to develop addictive behaviors because they use them frequently, which may harm a person's mental, social, and emotional health as well as cause behavioral and psychological issues (He and Yang 2022; Wang et al. 2015). According to previous findings, people with increased Facebook addiction had a higher prevalence of disordered eating behaviors and lower levels of satisfaction with their appearances (Mabe et al. 2014; Stronge et al. 2015). In addition, exposure to mass media is significantly affecting eating attitudes and behaviors in developing nations (Eapen et al. 2006; Musaiger et al. 2013). Moreover, video content on different types of foods is increasing day by day and influencing most of the young generations on their food preferences. Future studies are suggested to examine the relationship between addictive behaviors with various forms of eating habits and food preferences. Awareness programs about the risks of Facebook addiction—particularly among young people—can encourage more responsible use of social media, support healthy eating and lifestyle habits, and avert disordered eating behavior.

### 4.4 Other Notable Findings of this Study

Other behavioral factors, such as smoking, were also significantly associated with a higher risk of ED among young adults compared to non‐smokers. This result is similar to a previous study conducted in Pakistan, which showed ED was more likely associated with an individual's smoking habits than those who do not smoke (Mushtaq et al. 2023). The link between smoking and ED could be attributed to the perspective that nicotine is an agent both of stress reduction and weight control, but with long‐term implications for health risks (Solmi et al. 2016; White 2012). Impulsivity and body image concerns are shared psychological factors that could also lead people to both behaviors (Anzengruber et al. 2006). A mass awareness and social campaign is needed to prevent smoking and tobacco use among young adults.

Our study found that physically inactive participants were more likely to be at risk for ED than those who were active (regular physical activity). This result is consistent with a previous study (Grilo et al. 2021). According to previous research, adults who were physically inactive had more food cravings with higher BMI and less self‐control than adults who were more physically active (Shook et al. 2015). Further research is recommended to explore the relationship between physical inactivity with ED risk. Additionally, social awareness about the benefits of a healthy lifestyle should be increased to reduce sedentary lifestyles.

Results showed a statistically significant correlation between ED risk and self‐rated BMI status. Overweight and obese young people had a higher chance of developing an ED problem compared to their normal‐weight counterparts. Previous studies have shown similar results (Alhazmi and Al Johani 2019; Banna et al. 2021; Memon et al. 2012). Previous research in the United States showed that university students who were overweight or obese were more likely to be on diets because they wanted to lose weight than their normal‐weight friends (Desai et al. 2008). Moreover, disordered eating is more common among adolescents and young adults who are overweight (Desai et al. 2008). This highlights how important it is to look at BMI when assessing and dealing with ED symptoms in young adults.

### 4.5 Strengths and Limitations

The present study was the first to measure the relationships between problematic Facebook use and the risk of ED. This study focused on young adults, who are more vulnerable to Facebook addictions and unhealthy eating practices. It can be used as baseline evidence to adapt the required strategies to reduce unhealthy eating attitudes and behaviors. The study has some constraints as well. It was a cross‐sectional study (that means causal interference is limited), and most of the participants were students. Another limitation was the exclusive measurement of Facebook addiction, which does not accurately represent the general addiction to social media or the addiction to other online media, including Google, Instagram, X, and TikTok. Future studies should look at more subtypes of online addiction to better understand the prevalence of social media addiction among Bangladeshi young people. It would be more reliable if we could assess some other addictive behaviors with a risk of ED among young adults. Furthermore, the sample size was not nationally representative. The data were collected through self‐report, which implies that respondents may not have completed the questions truthfully. An additional limitation was the use of self‐reported data to assess BMI status (i.e., participants were asked to classify themselves as underweight, normal weight, or overweight/obese) rather than direct anthropometric measurements, which may have led to erroneous estimations.

## 5 Conclusion

In this study, almost one‐fourth of young adults were at risk of ED, and the prevalence of Facebook addiction was higher compared to previous studies in Bangladesh. The findings revealed that smoking habits, overweight and obesity, being physically inactive, and Facebook addiction were found to be potential risk factors for causing ED among young adults. Future longitudinal or qualitative studies should be conducted to identify whether other addictive behaviors as well as mental health disorders are also contributing to the development of ED. These results also highlight the need for an awareness campaign and an educational program to improve eating attitudes and behaviors. This may help public health professionals, policymakers, and health care professionals to take the initiative and develop strategies to overcome these addictive behaviors and promote a healthy lifestyle, as the Facebook addiction among the young generation is increasing with its effect on healthy dietary habits.

## Author Contributions

**Trisha Mallick**: conceptualization, software, formal analysis, investigation, methodology, writing–original draft. **Md. Hasan Al Banna**: conceptualization, writing–review and editing, methodology, writing–original draft. **Tasnim Rahman Disu**: writing–review and editing. **Shammy Akter**: writing–review and editing. **Tareq Mahmud**: investigation, data curation. **Tasnima Akhter Tasin**: investigation, data curation. **Nargees Akter**: investigation, data curation. **Md. Saiful Alam**: investigation, data curation. **Md. Nazmul Hassan**: visualization, validation, supervision.

### Peer Review

The peer review history for this article is available at https://publons.com/publon/10.1002/brb3.70540

## Data Availability

The data that support the findings of this study are available from the corresponding author upon reasonable request.
